# Living on the edge: stress and activation of stress responses promote lifespan extension

**DOI:** 10.18632/aging.100133

**Published:** 2010-04-09

**Authors:** Alice Zuin, David Castellano-Esteve, José Ayté, Elena Hidalgo

**Affiliations:** Oxidative Stress and Cell Cycle Group; Universitat Pompeu Fabra; 08003 Barcelona, Spain

**Keywords:** MAP kinase, aging, oxidative stress, protein kinase A, Sty1, Sck2

## Abstract

Oxidative stress constitutes the basis of physio-pathological situations such as neurodegenerative diseases and aging.  However, sublethal exposure to toxic molecules such as reactive oxygen species can induce cellular responses that result in stress fitness.  Studies in Schizosaccharomyces pombe have recently showed that the Sty1 MAP kinase, known to be activated by hydrogen peroxide and other cellular stressors, plays a pivotal role in promoting fitness and longevity when it becomes activated by calorie restriction, a situation which induces oxidative metabolism and reactive oxygen species production.  Activation of the MAP kinase by calorie restriction during logarithmic growth induces a transcriptional anti-stress response including genes essential to promote lifespan extension.  Importantly enough, the lifespan promotion exerted by deletion of the pka1 or sck2 genes, inactivating the two main nutrient-responsive pathways, is dependent on the presence of a functional Sty1 stress pathway, since double mutants also lacking Sty1 or its main substrate Atf1 do not display extended viability.  In this Research Perspective, we review these findings in relation to previous reports and extend important aspects of the original study.  We propose that moderate stress levels that are not harmful for cells can make them stronger.

Aging and lifespan extension have been a matter of debate for decades, with huge social interest in the civilized world, and much personal and financial effort focusing on this hot topic.  The molecular mechanisms that govern cellular aging have been conserved over the course of evolution, so that pluricellular and unicellular model systems share similar environmental and genetic strategies for modulating the aging process.  Several reports have indicated earlier that either calorie restriction or the inactivation of nutrient-dependent pathways (i.e. protein kinase A) is able to promote life extension in different eukaryotes.

In unicellular fungi, researchers use two different cellular situations in order to study the mechanisms of aging: replicative aging refers to the number of descendents that a cell can generate before its death, whereas chronological lifespan measures the viability of cultures at the stationary phase of the growth curve.  Therefore, chronological aging constitutes a model for differentiated somatic cells.  Recently, *Schizosaccharomyces pombe* has been used as a model system for the study of chronological aging.  As described for other eukaryotes, fission yeast cultures grown under low glucose conditions survive longer at the stationary phase than cultures grown in the same medium but with higher concentrations of the carbon source. It is worth pointing out that in both types of medium the concentration of glucose in the extracellular environment is undetectable soon after reaching the stationary phase.  Therefore, the type of metabolism occurring  -during  the metabolically  -active logarithmic cultures seems to condition chronological aging.  What is the link between calorie restriction and lifespan extension? When comparing *S. pombe *cultures growing in yeast extract-based media with 1% *versus* 4% glucose, we have determined that the respiratory rates differ considerably [1].  Indeed, low glucose cultures display significantly higher oxygen consumption levels, as an indicator of oxidative metabolism, than those of high glucose cultures.  Intracellular production of reactive oxygen species (ROS) is also more elevated in cells grown under low glucose conditions.  Under this situation, the MAP kinase Sty1, which is also a sensor of extracellular hydrogen peroxide stress (H_2_O_2_), becomes activated to a much higher extent in cells grown in this respiratory-prone medium, probably as a consequence of elevated ROS levels.  Since its identification in 1995 by Shiozaki, Russell and Millar groups [2,3], this MAP kinase has been traditionally linked to the activation of wide transcriptional responses promoting survival under diverse environmental stresses (for reviews, see [4,5]).  The activation of Sty1 at the onset of stationary phase only under conditions of calorie restriction suggests that the gene response triggered by this stressful situation may contribute to the establishment of a quiescent state which would allow survival under a hypometabolic stage.  In fact, cells lacking Sty1 or its main effector, the transcription factor Atf1 [6-8], display a compromised viability even under calorie restriction (Figure 1).  We believe that growth under low-glucose media promotes respiration *versus *fermentation, ROS production, Sty1 phosphorylation/ activation and as a consequence the induction of a transcriptional stress program which will contribute to the fitness of cells under starvation conditions (Figure 1).

**Figure 1.**
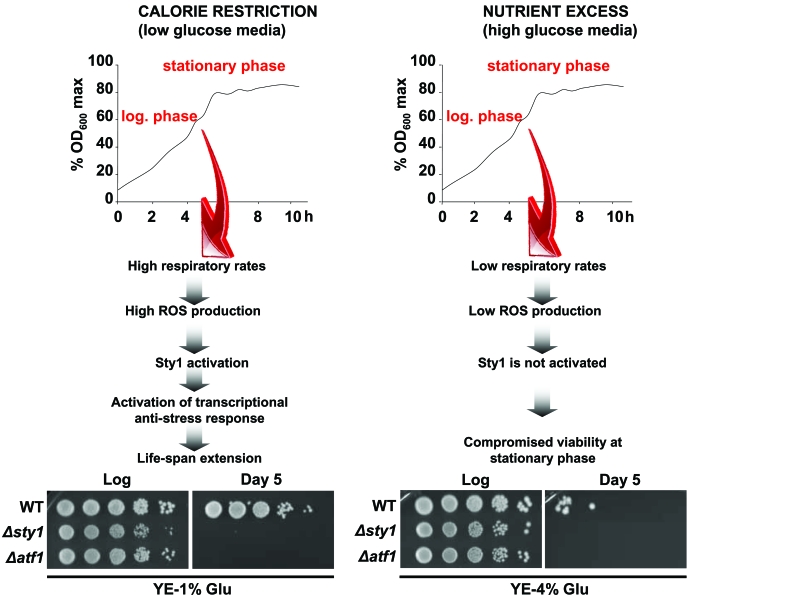
Activation of Sty1 stress response pathway is required for life extension upon calorie restriction. Scheme depicting the role of the Sty1 pathway on life-span promotion (see text for details). Strains 972 (WT), AV18 (*Δsty1*) and AV15 (*Δatf1*) were grown in YE-1% glucose media (calorie restriction condition) and YE-4% glucose media (glucose-rich conditions).  At the logarithmic phase (Log) or 120 hours after reaching the stationary phase (Day 5) serial dilutions of the cultures were plated onto YE plates.

In the process of chronological aging in fission yeast, we suggest that oxidative stress is exerting two antagonistic roles.  On one hand, during late logarithmic phase, we report the first side, a beneficial, signalling role of ROS: growth under calorie restriction allows for the activation of a ROS-activated, MAP kinase-driven signalling pathway which promotes a global transcriptional change (up to 400 genes can be regulated by Sty1) [9,10], meant to induce cellular fitness.  This hormetic effect of mild stresses, able to induce adaptive responses, has been widely reported in several model systems [11-15],  and the blockage  of such  non-toxic stress, for instance with antioxidants, may preclude its health-promoting effects [16].  On the other hand, death at the stationary phase may well be dependent on oxidative stress, as suggested by Rokeach and colleagues [17] and by ourselves [1]: the levels of ROS of live cells at stationary phase are higher in cultures from glucose-rich media (Figure 2A), as are the levels of carbonylated proteins (Figure 2B).  We suggest that, as widely reported in the literature (for a review, see [18]), oxidative stress is the main cause of the molecular damage associated with death in chronological aging.

**Figure 2.**
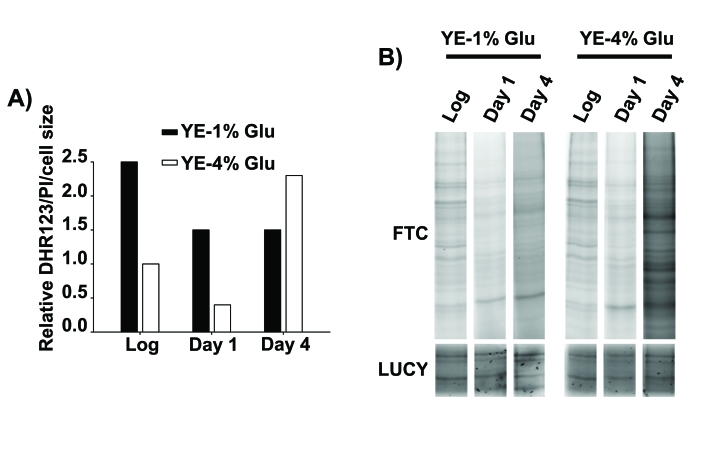
Oxidative stress as a cause of death of stationary phase, glucose-rich cultures. (**A**) Relative intracellular H_2_O_2_ levels of cells in logarithmic and stationary phase conditions. Wild type cells were grown in YE-1% and YE-4% glucose media.  At the logarithmic phase (Log) and one or four days after reaching stationary phase (Day 1 and Day 4) cells were incubated with the redox-sensitive dye dihydrorhodamine 123 (DHR123) and with the permeability-dependent dye propidium iodide (PI), and the fluorescence of live cells was analyzed by flow cytometry. The DHR123 green fluorescence was normalized to the PI red fluorescence and to the cell size (y axis: Relative DHR123/PI/cell size), and all the values are referred to that of YE-4% glucose culture in logarithmic phase, with an assigned value of 1. (**B**) Protein carbonylation generated during stationary phase in calorie restriction and rich glucose condition.  Cells from the same strains as in **A** were collected, protein samples were loaded in a SDS-PAGE gels and protein carbonylation was detected by fluorescein-5-thiosemicarbazide (Fluka-Sigma) fluorescence (FTC, top panel).  Protein carbonylation detection method was performed like in [34] with minor modifications. LUCY (Sigma) staining of total proteins was performed as a loading control (LUCY, bottom panel).

**Figure 3.**
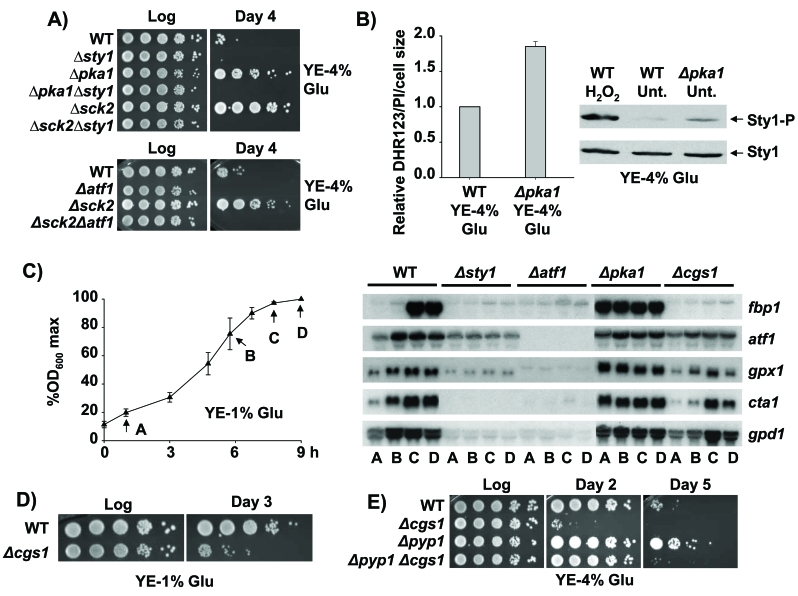
Role of the Sty1, Pka1 and TOR-Sck2 pathways in stationary phase. (**A**) Lack of Pka1 and Sck2 kinases promotes stationary phase cell survival under glucose rich conditions in a Sty1-, Atf1-dependent manner.  Strains 972 (WT), AV18 (*Δsty1*), MC22 (*Δpka1*), MC24 (*Δpka1 Δsty1*), MC25 (*Δsck2*), MC27 (*Δsck2Δsty1*), AV15 (*Δatf1*) and AZ118 (*h^-^ sck2::kanMX6 atf1::natMX6; Δsck2Δatf1*) were grown in YE-4% glucose media.  Serial dilutions of the logarithmic (Log) and stationary phase (Day 4) cultures were spotted onto YE plates.  (**B**) Loss of function of the glucose dependent Pka1 kinase triggers enhanced intracellular H_2_O_2_ levels and Sty1 activation.  Strains 972 (WT) and MC22 (*Δpka1) *were grown in YE-4% glucose media.  Cells were harvested at an OD_600_ of 0.5 and relative intracellular H_2_O_2_ levels were analysed as described in Figure 2A.  The same cultures were used to characterize Sty1 phosphorylation from TCA extracts, using anti-p38-P antibody.  Wild type cells treated with 1 mM H_2_O_2_ for 10 min (H_2_O_2_) were used as a control of activated Sty1.  Anti-Sty1 antibody was used as a loading control.  (**C**) Activation of the transcriptional stress response at stationary phase is Sty1-dependent and Cgs1-independent.  Strains 972 (WT), AV18 (*Δsty1*), AV15 (*Δatf1*), MC22 (*Δpka1*) and AZ106 (*h^-^ cgs1::kanMX6 ura4-D18; Δcgs1*) were grown in YE-1% glucose media. The time points of the five growth curve were recorded approximately at the same percentages of the maximum OD_600_ of each culture.  Standard deviation for every point is indicated.  At the time points indicated (A to D), cells were collected and RNA samples were obtained and hybridized against *fbp1*, *atf1*, *gpx1*, *cta1* and *gpd1*. (**D**) Pka1 pathway is required for stationary phase survival upon calorie restriction.  Strains 972 (WT) and AZ106 (*Δcgs1*) were grown in YE-1% glucose media.  At the logarithmic phase (Log) or 72 hours after reaching the stationary phase (Day 3) serial dilutions of the cultures were plated onto YE plates.  (**E**) Strains 972 (WT), AZ106 (*Δcgs1*), AZ103 (*Δpyp1*) and AZ115 (*h^-^ pyp1::kanMX6 cgs1::natMX6; Δpyp1 Δcgs1*) were grown in YE-4% glucose media.  At the logarithmic phase (Log) or several days after reaching stationary phase (Day 2 and Day 5) serial dilutions of the cultures were plated onto YE plates.

For any model system studied, it is widely accepted that the de-repression of pathways which should only be active upon calorie restriction is a genetic intervention which promotes lifespan extension (for reviews, see [19-22]).  For instance, both in budding and fission yeasts, deletion of the genes coding for the protein kinase A or the TOR  kinase substrate,  SCH9 (*S. cerevisiae*) / Sck2 (*S. pombe*) kinases, induces longevity even under glucose-rich conditions [1,17,23-25] (Figure 3A).  Is this genetically-driven lifespan promotion in any way connected to the Sty1 MAP kinase pathway in fission yeast? Apparently so, because cells carrying double deletions of the genes *pka1 *or*sck2, *coding for two kinases governing the two main nutrient-dependent pathways, and either the *sty1* or the *atf1 *genes [1] (Figure 3A), display a highly compromised viability at stationary phase.  We have reported that deletion of the *pka1 *gene leads to an enhanced oxygen consumption even with high glucose levels [1], elevated intracellular ROS (Figure 3B) and basal Sty1 phosphorylation [1] (Figure 3B), and this promotes cell survival without the need of calorie restriction-driven hormotic activation of stress responses.  In the case of the TOR substrate, Sck2, we suspect that deletion of its gene may also induce a subtle de-repression of respiration as it has been reported for the budding yeast homolog SCH9 [26], although we have not been able to experimentally probe it yet.

It is important to point out that the glucose-dependent Pka1 pathway has been traditionally linked to the stationary phase in fission yeast (for a review, see [27]).

In fact, a number of genes such as *fbp1 *(coding for the gluconeogenesis regulatory protein fructose-1,6-bisphosphatase; [28]) are triggered at the onset of stationary phase in a Pka1-dependent manner.  During logarithmic growth, that is, in the presence of glucose, Pka1 kinase is fully active and phosphorylates and inactivates the transcription factor Rst2, which cannot trigger *fbp1* transcription. Upon glucose depletion, cAMP levels decrease, and the regulatory subunit of Pka1, Cgs1, is then free to interact with the kinase, inactivate it and trigger Rst2-dependent *fbp1 *transcription.  Therefore, whereas deletion of the *pka1*gene induces lifespan extension by de-repressing its gene expression program and activating Sty1 (Figure 3ABC), deletion of *cgs1 *leads to a severe phenotype under calorie restriction, like the one described for cells lacking Sty1 or Atf1 (Figure 3D).  That indicates, as previously suggested, that activation of gene responses by both the Sty1-Atf1 pathway and the Pka1/Cgs1-Rst2 pathways are required for survival at stationary phase.

**Figure 4.**
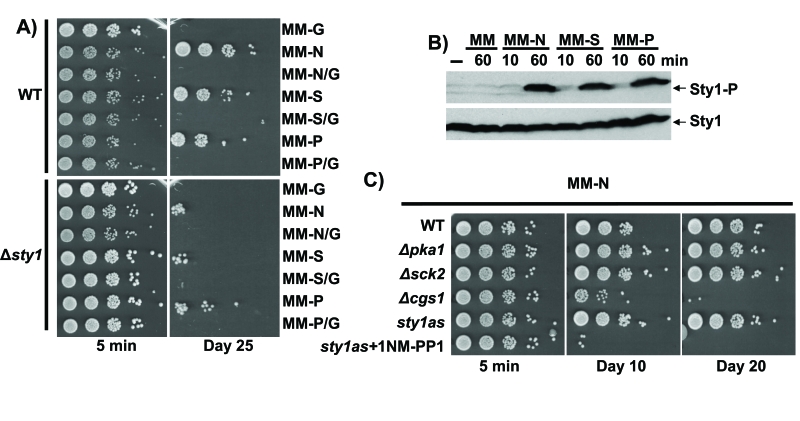
Quiescence establishment upon nitrogen, sulphate or phosphate starvation is glucose- and Sty1-dependent. (**A**) Sty1 and carbon source are necessary for lifespan extension upon nutrients starvation. 972 (WT) and AV18 (*Δsty1*) strains grown in minimal media until OD_600_ 0.5 were harvested by centrifugation. Then, cells were washed twice with minimal media without nutrients and resuspended to a final OD600 of 0.1 in minimal media lacking glucose (MM-G), nitrogen (MM-N), nitrogen and glucose (MM-N/G), sulphate (MM-S), sulphate and glucose (MM-S/G), phosphate (MM-P), phosphate and glucose (MM-P/G). Five minutes (5 min) and 25 days (Day 25) after the shift, serial dilutions of the cultures were plated onto YE plates.  (**B**) Sty1 is activated upon nutrients depletion.  Wild type cells treated like in **A** and resuspended in MM, MM-N, MM-S and MM-P with a final OD600 of 0.5 were recollected 10 or 60 minutes after the shift and Sty1 phosphorylation was determined from TCA extracts using anti-p38-P antibody.  Anti-Sty1 antibody was used as a loading control. (**C**) Lack of Cgs1 impairs quiescence maintenance upon nitrogen depletion. Strains 972 (WT), AZ74 (*h- pka1::kanMX6*), AZ73 (*h- sck2::kanMX6*), DC3 (*h- cgs1::kanMX6*) and AZ107, harbouring the as mutation *sty1*.T97A [1] and treated or not with 5µM ATP analogue 1NM-PP1 (*sty1as* + 1NM-PP1 and *sty1as* respectively) were grown in minimal media until OD_600_ 0.5. Addition of the ATP analogue specifically inhibits the Sty1.T97A kinase activity. Cells were harvested by centrifugation, washed twice with milli Q water and resuspended to a final OD_600_ of 0.1 in minimal media lacking nitrogen (MM-N). Five minutes (5 min), 10 and 20 days (Day 10 and Day 20) after the shift/resuspention serial dilutions of the cultures were plated onto YE plates.

Activation of *fbp1 *and other genes depends on both the Pka1 and the Sty1 pathways [29], whereas activation of the stress genes *atf1, gpx1, cta1 *and* gpd1 *depends mainly on the presence of Sty1 and Atf1 (Figure 3C).  We also know now that the activation of the MAP kinase dependent transcriptional response has a more prominent role than the one of the Pka1 pathway, since constitutive activation of Sty1 (by deletion of the gene coding for the Sty1 phosphatase Pyp1) can partially overcome the defects of cells lacking Cgs1, at least at early times (Figure 3E; Day 2); on the contrary, in the *∆pka1∆sty1* strain the phenotype of the *sty1 *deletion predominates (Figure 3A) [1].

In fission yeast, an experimental approach to study proliferation *versus *quiescence is to nutritionally starve logarithmically growing cultures by simply harvesting cells from complete media and re-suspending them in media depleted of an essential growth component.  The genetic bases for entry into and maintenance of quiescence upon nitrogen deprivation have been recently characterized [30-32], and we have observed that lack of phosphate or sulphate can also trigger viability in fission yeast (Figure 4A).  It is important to point out that, in these types of abrupt starvation, extracellular glucose cannot be depleted, suggesting that during logarithmic growth cells do not accumulate any energy source reservoir and that quiescent cells remain metabolically active [30] (Figure 4A).  Are the Sty1/Atf1 and the Pka1/Cgs1 pathways essential to promote entry into and maintenance of quiescence using this experimental approach?  Indeed, they are.  In a genetic screen to detect genes required for entry into and maintenance of quiescence upon nitrogen deprivation, strains lacking Sty1 or its double MAP kinase Wis1 were consistently isolated [31].  We have determined that the MAP kinase is also required to promote viability upon sulphate and phosphate starvation (Figure 4A).  Whatever the mechanism of activation may be, the MAP kinase becomes phosphorylated/activated by nitrogen [8], sulphate and phosphate depletion (Figure 4B). Importantly, gene induction by the Pka1 pathway may also be required to maintain quiescence, since cells lacking Cgs1 lose viability under nitrogen starvation (Figure 4C).

In conclusion, using fission yeast as a model system we confirm that moderate levels of stress due to oxidative metabolism during the logarithmic growth may prepare cells to encounter future periods of starvation or inactivity, and that a MAP kinase pathway has an essential role in linking endogenous stress and the activation of a genetic fitness program.  Similarly, a role for the Sty1 mammalian ortholog p38 in promoting senescence has been established (for a recent review, see [33]).  In fact, it has also been postulated that the beneficial effect on replicative aging of human fibroblasts of heat shock-induced hormesis is concomitant to enhanced levels of some MAP kinases [15].  Whether calorie restriction may exert a beneficial effect on human cells through activation of basal p38 activity remains to be demonstrated.

NOTE: Most of the experimental procedures, media and strains used to perform the figures in this manuscript are fully described in reference [1].  Only the strains generated for this work are described in the figure legends (complete genotypes in brackets).
